# Analysis of cell‐free fetal DNA for non‐invasive prenatal diagnosis in a family with neonatal diabetes

**DOI:** 10.1111/dme.13180

**Published:** 2016-07-31

**Authors:** E. De Franco, R. Caswell, J. A. L. Houghton, V. Iotova, A. T. Hattersley, S. Ellard

**Affiliations:** ^1^ Institute of Biomedical and Clinical ScienceUniversity of Exeter Medical SchoolExeterUK; ^2^ University Hospital ‘St. Marina’VarnaBulgaria

## Abstract

**Aims:**

An early genetic diagnosis of neonatal diabetes guides clinical management and results in improved treatment in ~ 40% of patients. In the offspring of individuals with neonatal diabetes, a prenatal diagnosis allows accurate estimation of the risk of developing diabetes and, eventually, the most appropriate treatment for the baby. In this study, we performed non‐invasive prenatal genetic testing for a fetus at risk of inheriting a paternal *KCNJ11* p.R201C mutation causing permanent neonatal diabetes.

**Methods:**

A droplet digital polymerase chain reaction assay was used to detect the presence of the mutation in cell‐free circulating DNA (cfDNA) extracted from maternal plasma at 12 and 16 weeks’ gestation.

**Results:**

The mutation was not detected in the cfDNA samples, suggesting that the fetus had not inherited the *KCNJ11* mutation. The fetal DNA fraction was estimated at 6.2% and 10.7%, which is above the detection limit of the assay. The result was confirmed by Sanger sequencing after the baby's birth, confirming that the baby's risk of developing neonatal diabetes was reduced to that of the general population.

**Conclusions:**

We report the first case of non‐invasive prenatal testing in a family with neonatal diabetes. A prenatal diagnosis in families at high risk of monogenic diabetes informs both prenatal and postnatal management. Although the clinical impact of this novel technology still needs to be assessed, its implementation in clinical practice (including cases at risk of inheriting mutations from the mother) will likely have a positive impact upon the clinical management of families affected by monogenic diabetes.

## What's new?

- This article describes the first case of non‐invasive prenatal diagnosis using cell‐free fetal DNA in a family with neonatal diabetes.
- The test showed that the baby had not inherited the neonatal diabetes mutation, providing reassurance to the parents at mid‐gestation that this baby was not at high risk of neonatal diabetes and informing management of the pregnancy, delivery and neonate accordingly.

**What's new?**

- This article describes the first case of non‐invasive prenatal diagnosis using cell‐free fetal DNA in a family with neonatal diabetes.
- The test showed that the baby had not inherited the neonatal diabetes mutation, providing reassurance to the parents at mid‐gestation that this baby was not at high risk of neonatal diabetes and informing management of the pregnancy, delivery and neonate accordingly.

## Introduction

Neonatal diabetes is diagnosed before 6 months of age and affects ~ 1 in 100 000 live births 1. Almost 40% of people affected with neonatal diabetes have mutations in the genes encoding for the subunits of the ATP‐sensitive potassium channel, *ABCC8* and *KCNJ11*. Approximately 20% of individuals with a mutation in a K_ATP_ channel gene have additional neurological features, most commonly developmental delay and, in the more severe cases, early‐onset epilepsy 2, 3. A recent study suggested that all individuals with neonatal diabetes caused by mutations in *ABCC8* or *KCNJ11* have some degree of neurological impairment 4. Most of these individuals can be successfully treated with sulfonylurea instead of insulin, leading to improved glycaemic control and neurological function 5, 6. Current evidence indicates that the earlier sulfonylurea treatment is started, the better the outcome for neurological function 7, 8. An early genetic diagnosis of neonatal diabetes is, therefore, essential to guide treatment decisions, with benefits for the management of both diabetes and additional complications 9.

Analysis of the cell‐free fetal DNA (cffDNA) present in maternal blood provides a non‐invasive means of testing pregnancies at high risk of genetic conditions. It has transformed prenatal genetic testing for Down syndrome 10. cffDNA is present in the maternal circulation from the first trimester at a proportion of ~ 10% of the total circulating DNA 11. The implementation of highly sensitive techniques such as massively parallel deep sequencing 12 and droplet digital polymerase chain reaction (ddPCR) 13 allows the detection of variants that are present in the fetus, but not in the maternal DNA (either paternally inherited or *de novo*) with high reliability.

In this report, we describe the use of a ddPCR assay for non‐invasive prenatal testing in the fetus of a couple in which the father has permanent neonatal diabetes due to a heterozygous *KCNJ11* p.R201C mutation.

## Patients and Methods

Informed consent was obtained from the couple. Cell‐free DNA (cfDNA) was extracted from maternal plasma collected at 12 and 16 weeks’ gestation using the Chemagic Circulating NA kit (Perkin‐Elmer, Waltham, MA, USA). Detection of the *KCNJ11* c.601C>T, p.R201C mutation (NM_000525.3) was carried out by ddPCR. A custom Life Technologies (Carlsbad, CA, USA) TaqMan assay was designed to detect the C (reference) and T (mutated) alleles: forward PCR primer, CGGCCGCCTCTGCTT; reverse PCR primer, TCATGCTCTTGCGGAGGTC; probe 1 (C allele), VIC‐ACCCACACGTAGCATG‐NFQ; probe 2 (T allele), FAM‐CCCACACATAGCATG‐NFQ. This assay was used in reactions for droplet generation, PCR and detection using the Bio‐Rad (Hercules, CA, USA) QX200 ddPCR system, as recommended by the manufacturers, with an annealing/extension temperature during PCR of 55°C. All data were analysed using Bio‐Rad QuantaSoft software, with merging of data for multiple wells where relevant.

Assays were performed on paternal genomic DNA (gDNA) (known genotype C/T), maternal gDNA (known genotype C/C) and cfDNA extracted from maternal plasma (genotype and gender unknown). To establish the sensitivity of the assay for the T allele in a background of maternal gDNA, control samples were prepared in which paternal gDNA was spiked into maternal gDNA at concentrations of 10%, 1% and 0.1% of total DNA.

To establish the proportion of cffDNA in the cfDNA samples from maternal plasma, parental genomic DNA samples were assayed by ddPCR using a panel of common single nucleotide polymorphisms to identify one for which the fetus would be an obligate heterozygote. The single nucleotide polymorphism selected was rs4536103 (*NEUROG3*, NM_020999.3:c.596T>C) at which mother and father were homozygous for the A or G alleles, respectively. Thus, the presence of the paternally inherited G allele in the cfDNA sample allowed accurate estimation of the percentage of cffDNA in the 12‐ and 16‐week samples.

## Results

**Figure 1:**
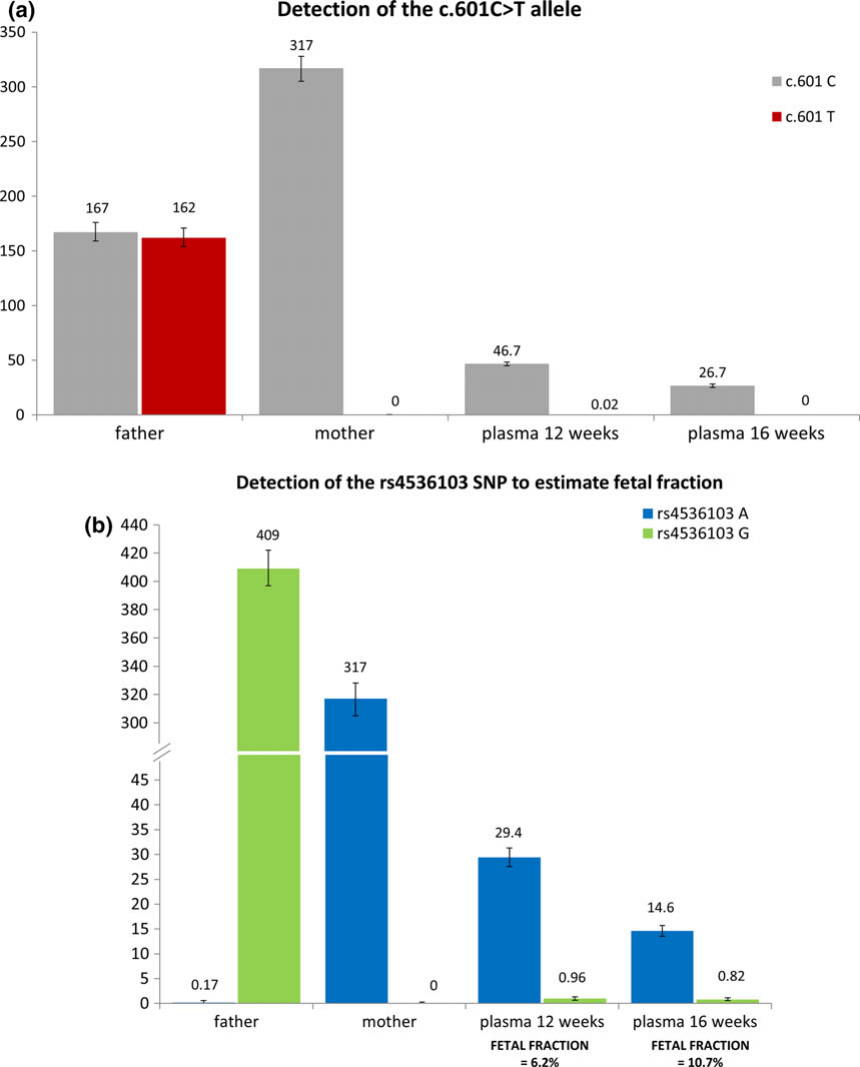
(a) Allele concentration for the *KCNJ11* c.601C>T mutation. The graph shows data for DNA samples from father, mother, 12 weeks cfDNA and 16 weeks cfDNA, as labelled. Grey bars represent the C allele; red bars represent the T allele (mutated). The *y*‐axis shows allele concentration (copies/μl, as reported by QuantaSoft software) on a linear scale, with data labels showing allele concentrations for individual samples; error bars show 95% Poisson confidence limits, as calculated by QuantaSoft. Total numbers of droplets analysed were: paternal gDNA, 11 808; maternal gDNA, 12 798; 12 weeks cfDNA sample, 74 635; 16 weeks cfDNA sample, 48 168. (b) Estimation of fetal fraction from the allele concentration of the single nucleotide polymorphism rs4536103. The same samples shown in (a) were assayed for the single nucleotide polymorphism rs4536103 (blue bars represent A allele, green bars represent G allele). Allele concentration (copies/μl) is shown on the *y*‐axis using a linear scale. Data labels above each bar show the allele concentration in individual samples; error bars show 95% Poisson confidence limits. Total numbers of droplets analysed were: paternal gDNA, 13 774; maternal gDNA, 12 371; 12 weeks cfDNA sample, 39 163; 16 weeks cfDNA sample, 54 576. Estimated fetal fraction in cfDNA, calculated as [(*n* copies of paternal allele × 2)/total *n* copies]%, is indicated under the graph.

Counts of the C (reference) and T (mutated) alleles for the *KCNJ11* c.601C>T mutation in paternal and maternal gDNA using ddPCR were consistent with the known genotypes of these samples (father heterozygous C/T, mother homozygous C/C), with paternal alleles present at a 50:50 ratio (within 95% Poisson confidence intervals) as expected (Fig. 1a).

The sensitivity of the assay was assessed using maternal gDNA spiked with paternal gDNA at various concentrations. The fraction of fetal DNA in maternal cfDNA is typically in the range 2–20% 11, and we were able to readily detect the paternal T allele when spiked into maternal gDNA at a concentration of 1% (data not shown). We set a conservative limit for reliable detection of the T allele as 10 positive droplets across all wells analysed (26 881 droplets in total for the 1% spiked sample), which equates to ~ 18 copies of amplifiable DNA. No droplets positive for the T allele were observed in negative (no template) controls or in maternal gDNA samples. Analysis of cfDNA extracted at either 12 or 16 weeks’ gestation showed that although the C allele was abundant, the T allele was not reliably detected. Pooled data from replicate wells gave an apparent concentration of 0.016 copies/μl in the 12‐week sample (one positive droplet observed of 74 635 analysed), whereas no positive droplets for the T allele were observed in the 16‐week sample (Fig. 1a). These results suggest that the fetus had not inherited the *KCNJ11* p.R201C mutation.

Estimation of the fetal DNA fraction in the two cfDNA samples using rs4536103 (for which the fetus was an obligate heterozygote) confirmed the presence of cffDNA at a relative amount of 6.2% in the 12 weeks’ gestation sample and 10.7% in the 16 weeks’ sample, consistent with previous reports 11 (Fig. 1b).

Given the fetal fractions measured using rs4536103, had the fetus inherited the paternal *KCNJ11* c.601T allele this would have been expected at an abundance of ~ 173 copies or ~ 114 copies in the ddPCR assay. Because these values are well above the limit of sensitivity, this confirms that absence of the mutant allele was due to non‐inheritance and not a failure to detect the allele as a result of low fetal fraction. The risk of the baby developing neonatal diabetes was, therefore, reduced to that of the population, and non‐inheritance of the *KCNJ11* p.R201C mutation was confirmed by direct Sanger sequencing of a DNA sample extracted from cord blood soon after the baby's birth.

## Discussion

We describe the application of a ddPCR assay for non‐invasive prenatal genetic testing in the offspring of a father heterozygous for a *KCNJ11* p.R201C mutation causing neonatal diabetes. Our assay showed that the fetus had not inherited the mutation and this result was confirmed after birth. This is the first report of prenatal diagnosis using cffDNA in a family with neonatal diabetes.

Early diagnosis of neonatal diabetes is essential to provide the most appropriate treatment, because most individuals with mutations in the potassium channel genes achieve improved glycaemic control upon switching from insulin to sulfonylurea therapy 5, 6. Furthermore, early treatment with high‐dose sulfonylureas is likely to improve neurological development in the 20% of individuals with neurological features. In the case we report, the fetus had not inherited the mutation and we could reassure the parents that the risk of developing neonatal diabetes was reduced to that of the population. This couple had sought prenatal testing in order to be prepared for the birth of a baby likely to develop diabetes during the first weeks of life. If the fetus had inherited the mutation, the baby's blood glucose would have been closely monitored and sulfonylurea therapy initiated promptly.

In this case, the father was affected with neonatal diabetes, so cffDNA analysis of the *KCNJ11* mutation was focused on detection of an allele that was not present in the maternal circulating DNA. Detection of mutations that are maternally inherited is more challenging because of the background maternal genome confounding the analysis. Improvement of cffDNA prenatal testing using highly sensitive techniques such as ddPCR may overcome this difficulty, allowing reliable testing for maternally inherited mutations. Prenatal testing is particularly relevant for the optimal management of diabetes in pregnant women with a mutation in one of the genes (*KCNJ11* or *ABCC8*) encoding the potassium channel subunits. Previous studies have shown that sulfonylureas can cross the placenta and influence fetal growth, potentially resulting in macrosomia and neonatal hyperinsulinaemia in babies who do not inherit the mutation 14.

Knowledge of the fetal genotype will influence both prenatal and postnatal management of pregnancies in which one parent is affected with neonatal diabetes. We illustrate how non‐invasive prenatal testing using cffDNA allowed accurate and timely diagnosis for a pregnancy at risk of neonatal diabetes due to a paternal mutation. Although the clinical impact of this novel technology needs to be formally assessed, we anticipate that its implementation in clinical practice (including cases at risk of inheriting maternal mutations) will likely provide clinical benefits for families affected by monogenic diabetes.

## Funding sources

ATH and SE are the recipients of a Wellcome Trust Senior Investigator award and ATH is employed as a core member of staff within the NIHR funded Exeter Clinical Research Facility. EDF is a Naomi Berrie Fellow in Diabetes Research.

## Competing interests

None declared.
